# The effect of a dietary supplement (N-oleyl-phosphatidyl-ethanolamine and epigallocatechin gallate) on dietary compliance and body fat loss in adults who are overweight: A double-blind, randomized control trial

**DOI:** 10.1186/1476-511X-11-127

**Published:** 2012-10-04

**Authors:** Gerald T Mangine, Adam M Gonzalez, Adam J Wells, William P McCormack, Maren S Fragala, Jeffrey R Stout, Jay R Hoffman

**Affiliations:** 1 Sport and Exercise Science, University of Central Florida, 4000 Central Florida Blvd, Orlando, FL 32816, USA

**Keywords:** Dietary Adherence, Appetite, Obesity, Green Tea Extract

## Abstract

**Background:**

A dietary supplement containing a blend of 170 mg of N-oleyl-phosphatidylethanolamine (NOPE) and 100 mg of epigallocatechin-3-gallate (EGCG) has been shown to improve compliance to low caloric diets. Considering the cost of dietary ingredients, many manufacturers attempt to determine the lowest efficacious dose. Thus, the purpose of this study was to evaluate the efficacy of 8-weeks of supplementation with a daily intake of 120 mg of NOPE and 105 mg of EGCG in conjunction with a low caloric diet and regular, moderate exercise on dietary compliance in healthy, overweight adults. An additional purpose was to examine the effect of this supplement/diet/exercise paradigm on changes in body composition, sensation of appetite, mood and severity of binge eating.

**Methods:**

Fifty healthy, overweight (BMI > 25 m·kg^2^) men (15) and women (35) (SUP; n = 25; 32.7 ± 13.75 y; BMI = 33.4 ± 6.2; PLA; n = 25, 34.3 ± 12.7 years; BMI = 33.2 ± 6.8) were recruited for a double-blind, placebo controlled study. Each volunteer was randomly assigned to either the supplement (SUP; n = 25) or placebo group (PLA; n = 25). Based upon a self-reported 3-day dietary recall all volunteers were recommended a 500 kcal or 30% (maximum of 1000 kcal) reduction in caloric intake. Volunteers were also encouraged to exercise 30 minutes per day, three times per week.

**Results:**

Subjects in SUP were significantly more compliant (*x*^2^ = 3.86, *p* = 0.049) in maintaining a low caloric diet at week 4, but this was not able to be maintained through the 8-week study. In addition, a significant difference in mood, feelings of fatigue and confusion were noted between the groups at week 4, but again not maintained by week 8 where only feelings of tension were improved. No differences between groups (*p* > 0.05) were observed for body mass, body composition, feelings of hunger, and binge eating after eight weeks.

**Conclusion:**

Supplementing with a combination of 120 mg of NOPE and 105 mg of EGCG does appear to enhance compliance to a low caloric diet and improve mood for 4 –weeks, but loses its effectiveness by week 8.

## Introduction

Excess body weight is a major health problem in the United States. More than 60% of adult Americans are classified as being overweight [1] and approximately 34% as obese [2]. Excess body fat increases the risk for several debilitating and potentially fatal diseases [3-7]. Research indicates that body mass index, waist circumference, and body fat percentages can be significantly reduced through a combination of diet and exercise, in comparison to diet alone [8,9]. However, a major determinant for success during a weight loss regimen is an individual’s ability to adhere to a low calorie diet for an extended duration [10,11]. Although caloric restriction is an effective method of losing body weight and body fat, it becomes more challenging if adherence to the diet cannot be maintained [11]. In response to this need the pharmaceutical industry developed a number of prescription medications to enhance the success of weight loss programs. Although there has been varying degrees of success [12], a high incidence of adverse events has shifted research to the development of non-prescription interventions [13-15]. Non-prescription weight loss supplements focus on several different mechanisms of action including enhancing satiety, thermo genesis, and the inhibition of enzymes that metabolize both carbohydrates and fats [16]. However, the efficacy and safety of these interventions has not been fully elucidated.

N-oleoyl-phophatidyl-ethanolamine (NOPE) and epigallocatechin gallate (EGCG) are naturally occurring nutritional ingredients, whose biological properties may enhance compliance to a reduced calorie diet [17-28]. NOPE, a naturally occurring phospholipid found in animal and vegetable foods, is hydrolyzed into N-oleyl-ethanolamide (NOE) and phosphatidic acid when consumed [17]. Its proposed mechanism of action is related to NOE’s role in inhibiting the effect of anandamide (N-arachidonyl-ethanolamine), an agonist of both peripheral and central cannabinoid type 1 receptors (CB1) whose activation leads to an increase in appetite and, consequently, an intake of food [18]. In rats, an intra-peritoneal injection of NOE has been shown to promote an anorexic effect through the activation of several intestinal receptors, which signal the brain center to reduce food intake [19,20]. EGCG, from standardized green tea extract, has been shown to stimulate weight [21,22] and fat loss [22,23] by inhibiting enzymes that contribute to the degradation of catecholamines [24,25], which stimulate lipolysis. In mice fed a high-fat diet, EGCG supplementation has been shown to attenuate gains in body mass, total body fat, and visceral fat [26]. Others have suggested that NOPE and EGCG by themselves may not be effective for weight loss [27,28], However, other investigators have demonstrated that a combination of these compounds may result in improved absorption of EGCG and prevent the rapid breakdown of NOPE, improving its availability [28].

A recent study in humans reported that a combination of NOPE (170 mg·day^-1^) and EGCG (100 mg·day^-1^) resulted in greater low calorie dietary compliance, improved mood and satiation, as well as decreases in depressive symptoms compared to a placebo [17]. However, weight loss was similar between groups, suggesting body weight reductions were not directly related to the dietary supplement. Thus, the purpose of this study was to determine if a proprietary blend of NOPE and EGCG, using lower concentrations of NOPE than previously reported, in conjunction with regular exercise, could improve dietary compliance to a low caloric diet and result in positive body composition changes in healthy, overweight adults.

## Methods

### Subjects

Fifty healthy adults (35 female, 15 male) volunteered to participate in an 8-week randomized, double-blind investigation. Following an explanation of all procedures, risks, and benefits associated with the experimental protocol, each volunteer gave his or her written informed consent to participate in this study. The research protocol was approved by the Institutional Review Board at the University of Central Florida. Inclusion criteria required participants to be between 18 – 59 years of age, with a body mass index (BMI) greater than 25 but less than 40, and their daily energy intake to be at, or above their calculated dietary fuel requirement. Participants were randomly assigned to one of two groups. One group was provided the supplement (SUP; n = 25; 17 females, 8 males; 32.7 ± 13.7 years; 93.3 ± 19.5 kg; BMI = 33.4 ± 6.2; 43.2 ± 7.2% Body Fat) containing 120 mg NOPE and 105 mg of EGCG, and the second group was provided a placebo (PLA; n = 25, 18 females, 7 males; 34.3 ± 12.7 years; 92.8 ± 18.6 kg; BMI = 33.26 ± 6.8; 44.0 ± 6.8% Body Fat) containing an equivalent amount of rice flour.

### Study design

**Figure 1:**
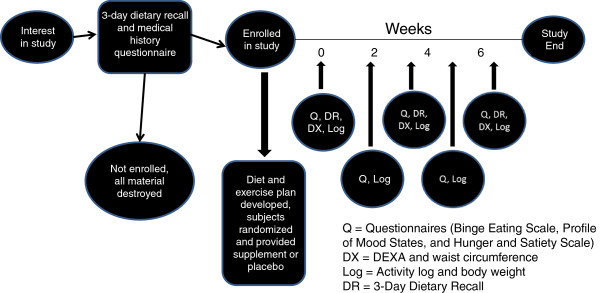
Flowchart of study design.

The study protocol was 8-weeks in duration and included a total of 6 visits with the investigators that included an initial familiarization visit. If enrolled, the subject returned to the Human Performance Laboratory for baseline measures (WK0) and then every two weeks for follow-up assessments (WK2, WK4, Wk6, and WK8, respectively). During the initial familiarization session, an explanation of study procedures associated with the experimental protocol was provided to potential subjects. Upon giving his or her written informed consent to participate in the study, the subject was asked to complete a medical history questionnaire. In addition, the subject’s BMI was calculated and then checked to determine if the subject met the investigation’s inclusion criteria. If the subject was enrolled into the study, they were then instructed to complete a 3-day dietary recall, consisting of two weekdays and one weekend day, to determine daily caloric intake. The 3-day dietary recall was returned for analysis prior to baseline measurements, to determine if caloric intake met the study’s inclusion criteria. During each subsequent laboratory visit anthropometric measures and study questionnaires were completed. The study protocol is described in Figure 1.

During the study subjects were recommended to maintain a regular exercise schedule that included at least 3 days per week, for 30 minutes per day at an intensity that that would elicit between 60 – 75% of the subject’s maximum heart rate. Volunteers were instructed to record their daily activity and provide these records to the investigators at each visit.

### Dietary analysis

The 3-day dietary recall was analyzed using the Food Works 13 Nutritional Assessment Software (The Nutrition Company, Long Valley, NJ, USA) and average caloric intake was determined. Total Energy Expenditure (TEE) was determined by a formula previously published [29] and used to exclude individuals who did not meet the study’s inclusion criteria. Subject’s TEE was determined by age, height, weight, and reported physical activity. If the subject’s dietary recall indicated that their daily caloric intake was at or above the TEE, they were then recommended a diet that was 30% or 500 kcal per day (whichever was greater) less than what they had been consuming. However, the maximum restriction did not exceed 1000 kcal per day. The same investigator provided dietary recommendations for each subject. Compliance was measured as the ability to maintain the recommended hypo-caloric diet over the course of the 8-week investigation. Caloric intake was reevaluated at WK4 and WK8.

### Anthropometric measurements

Anthropometric measurements for all participants were conducted in the following sequence: height (WK0 only) and body mass, body mass index (BMI), waist circumference, and Dual Energy X-Ray Absorptiometry (DEXA) measurements. Body mass (measured to the nearest 0.1 kg) and height (measured to the nearest 0.01 m) were determined using a Health-o-meter Professional (Patient Weighing Scale, Model 500 KL, Pelstar, Alsip, IL, USA). Measurements were performed with the participants standing barefoot, with feet together, in their normal daily attire. Body mass index (BMI) was calculated as measured weight divided by the square of measured height. Waist circumference was measured using techniques previously described [30] by the same investigator for each subject.

Body composition was determined using whole body-dual energy x-ray absorptiometry (DEXA) scans (Prodigy™; Lunar Corporation, Madison, WI). Total body estimates of percent fat, fat and non-bone lean tissue was determined using company’s recommended procedures and supplied algorithms. Quality assurance was assessed by daily calibrations and was performed prior to all scans using a calibration block provided by the manufacturer.

Abdominal fat was determined as previously described [31]. Briefly, a quadrilateral box was manually drawn around the L1-L4 region of interest (abdomen) bounded inferiorly by the horizontal line identifying the L4/L5 vertebral space and superiorly by the horizontal line identifying the T12/L1 vertebral space. Scans were displayed with an adjustment of the gray scale, so that all of the soft tissue in the designated area was included.

### Questionnaires

The Hunger and Satiety Scale (HSS) [32], Profile of Mood States (POMS) [33], and Binge Eating Scale (BES) [34] were administered during each visit beginning WK0. The HSS was assessed numerically, using a scoring system graded from 0, to represent ravenous hunger, to plus 10, which represents feeling stuffed or overfull. Volunteers were shown a scale with ten gradations and asked to indicate how they felt in respect to hunger or satiety by circling the appropriate place along the scale. The scale also consisted of phrases describing the various degrees of hunger or satiety, but volunteers were free to choose any point along the scale [32]. The POMS consists of 58 words or phrases in a Likert format questionnaire which provides measures of specific mood states. It provides measures of tension, depression, anger, vigor, fatigue and confusion. A total mood score is computed by subtracting vigor from the sum of the five other negative measures and adding 100 to avoid a negative result [33]. The BES, which has been shown to be a valid and reliable measure of classifying binge eaters, included sixteen items measuring the severity of binge eating. It examines both behavioral manifestations (eating large amounts of foods) and feeling/cognition during a binge episode (loss of control, guilt, and fear of being unable to stop eating). Participants scoring 17 or lower on the BES were classified as non-binge eaters, those with a score of 18 – 26 as moderate binge eaters, and those scoring 27 or higher as severe binge eaters [34]. All questionnaires were performed under controlled conditions (a quiet room alone with the investigator). During all test administrations participants were asked to describe their feelings upon how they were feeling at the moment.

### Supplement

The supplement, marketed as PhosphoLean™ (Chemi Nutra, White Bear Lake, MN), contained a proprietary blend of NOPE, a naturally occurring phospholipid found in soy, and EGCG, from standardized green tea extract. Each capsule contained 40 mg of NOPE, 35 mg of EGCG and 25 mg of mixed phospholipids. The placebo contained an equal amount of rice flour. Participants were instructed to ingest 300 mg per day (three capsules, 100 mg per capsule) of SUP or PLA (100 mg rice flour per capsule); one capsule was consumed 60 minutes prior to lunch and two capsules 60 minutes prior to dinner.

### Statistical analysis

The efficacy of the supplement was determined by calculating the change (∆ score) from WK0 to each measuring point (WK2, WK4, WK6 and WK8). Due to participant drop out or removal, the number of participants per visit was reduced. To account for the different sample sizes at each time point independent t-tests using the Bonferroni method to correct for multiple assessments was employed to compare group differences. Chi-square analysis was used to analyze compliance at WK4 and WK8. A criterion alpha level of p ≤ 0.05 was used to determine statistical significance prior to Bonferroni correction.

## Results

Thirty-two (SUP: 11 females, 5 males; PLA: 12 females, 4 males) of the original fifty volunteers completed the study. Seven participants (SUP = 4, PLA = 3) dropped out by WK2, Four additional participants (SUP = 2, PLA = 2) dropped out after WK4, and five participants (SUP = 3, PLA = 2) were deemed to be non-adherent. Non-adherence was defined as participants who did not take a minimum of 70% of the product or who did not complete their final measurements. Their data were not included in any of the analyses. Of the remaining two participants, one in the SUP group withdrew from the study (prior to WK2) when she was diagnosed with hypothyroidism, which was not related to the supplement. The other subject, in the placebo group, experienced blurry vision, headache, increased appetite, shakiness, and disrupted alertness prior to WK2. Analysis of dietary recalls indicated greater compliance (*x*^2^ = 3.86, *p* = 0.049) to the low caloric diet in SUP (57.14%) compared to PLA (42.86%) at WK4. However, no differences (*x*^2^ = 0.000, *p* = 1.000) in dietary compliance were noted between the groups by WK8. When comparing caloric intake between groups, no difference from the change from WK0 were seen between SUP (-889 ± 662 kcal and -911 ± 871 kcal) and PLA (-619 ± 1127 kcal and -718 ± 1337 kcal) at WK4 and WK8, respectively.

**Figure 2:**
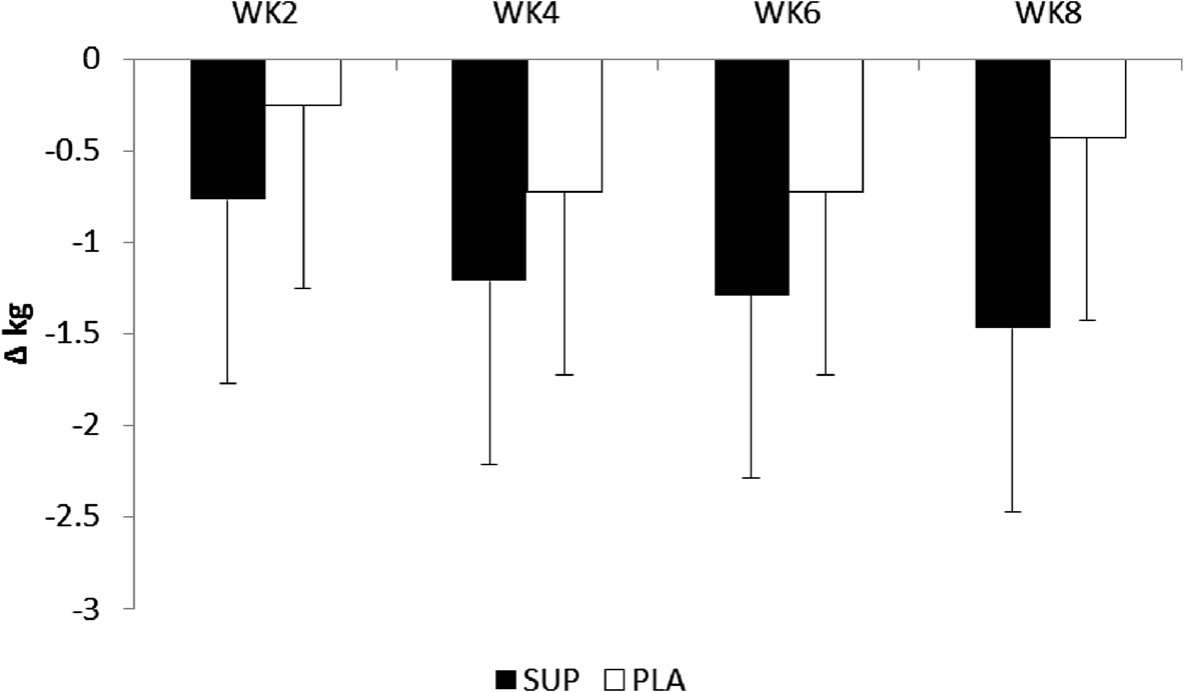
Changes from baseline in Body Mass.

Analysis of the participants self-reported exercise habit revealed no significant differences (p = 0.577) in weekly training frequency between PLA (p = 0.577; 2.7 ± 1.0 days per week) and SUP (3.0 ± 1.6 days per week) or in minutes per workout (p = 0.884; 55.6 ± 25.5 min versus 54.3 ± 15.5, respectively). No significant differences were seen between the groups in changes of body mass at any time point (see Figure 2). Similarly, no significant differences were noted in the change in % body fat (-0.4 ± 2.0% versus -0.1 ± 2.8%), lean body mass (-0.4 ± 1.6 kg versus -0.2 ± 1.6 kg), waist circumference (-3.7 ± 1.6 cm versus -0.3 ± 7.8 cm) and central adiposity (-0.3 ± 1.4 kg versus -0.3 ± 0.8 kg) between PLA and SUP, respectively.

**Figure 3:**
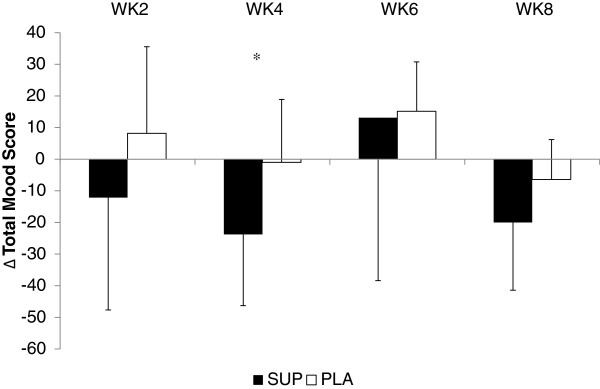
**Changes in total mood score.** * = Significant difference (p = 0.006) between groups.

Changes from WK0 in HES and BES scores are shown in Table 1. No significant differences in the ∆ scores between the groups were noted at any time point. The total mood score from the POMS assessment is depicted in Figure 3. A significant difference between the groups was seen at WK4. Participants ingesting the supplement had significantly (p = 0.006) greater decrease in total mood (a favorable response) than PLA. Similar patterns were also noted for fatigue (p = 0.012) and confusion (0.009) at WK4 (see Table 1). The only other significant difference noted was observed at WK8, where changes in tension was significantly (p = 0.002) different between the groups.

**Table 1:** Changes from WK0 in hunger and satiety, binge eating and profile of mood states questionnaires

		**WK2**	**WK4**	**WK6**	**WK8**
Hunger and Satiety Scale	PLA	0.21 ± 2.97	0.06 ± 2.28	0.46 ± 1.66	0.06 ± 1.39
	SUP	0.58 ± 1.39	0.27 ± 2.15	0.62 ± 1.71	0.31 ± 1.40
Binge Eating Scale	PLA	-4.74 ± 3.75	-6.53 ± 4.87	-8.00 ± 3.65	-7.06 ± 4.99
	SUP	-5.74 ± 6.88	-5.71 ± 6.68	-5.67 ± 5.79	-6.53 ± 7.15
**Profile of mood states**					
Tension	PLA	1.79 ± 5.90	1.35 ± 3.97	2.62 ± 7.70	0 ± 2.47
	SUP	-0.37 ± 3.40	-2.07 ± 4.08	-2.25 ± 4.92	-3.20 ± 2.76*
Depression	PLA	2.84 ± 8.17	0.88 ± 3.35	3.23 ± 11.58	0.59 ± 4.30
	SUP	-1.37 ± 2.34	-1.86 ± 2.93	-1.17 ± 3.10	-1.07 ± 3.45
Anger	PLA	2.42 ± 3.88	0.82 ± 1.94	2.38 ± 5.38	0.41 ± 1.33
	SUP	-0.53 ± 3.36	-1.21 ± 2.39	-0.58 ± 3.40	-0.40 ± 3.25
Vigor	PLA	1.79 ± 16.31	5.47 ± 17.85	-0.38 ± 15.88	2.59 ± 14.62
	SUP	3.47 ± 12.75	8.21 ± 11.37	4.17 ± 8.48	7.80 ± 9.79
Fatigue	PLA	-0.47 ± 8.58	0.35 ± 4.40	2.85 ± 10.64	-3.65 ± 6.26
	SUP	-4.84 ± 11.35	-5.64 ± 7.90*	-3.58 ± 5.43	-5.27 ± 6.30
Confusion	PLA	3.37 ± 8.22	1.06 ± 5.03	3.69 ± 10.73	-1.12 ± 5.17
	SUP	-1.58 ± 5.27	-4.79 ± 6.57*	-1.33 ± 5.99	-2.27 ± 55.11

* = significant difference between groups.

## Discussion

The results of this study suggest that a daily intake of 120 mg NOPE and 105 mg of EGCG for 4-weeks, can enhance compliance to a low caloric diet, total mood score, feelings of fatigue, and confusion, but does not alter body mass or body fat to any greater magnitude than placebo. However, these benefits were not maintained by week 8 of the study, where only feelings of tension were reduced in comparison to placebo. These findings contrast with Rondanelli et al., [17] in which sustained compliance and improvements in feelings of satiety, and severity of binge eating were observed for 8-weeks of study duration.

It is possible that the method used to determine dietary compliance may have contributed to the differences between the studies. In the present investigation, compliance was defined as the ability to maintain the recommended low caloric diet. Rondonelli and colleagues defined compliance in terms of drop-out rate [17]. This may not entirely capture compliance, as approximately 72% of the participants, who did not drop out of the present study, were also unable to maintain the low caloric diet at one or more time points. This is supported by research investigating the psychological biases in self-reporting dietary habits in studies of caloric restraint [35,36]. Although a participant may appear to be following the experimental protocol by not withdrawing, many may seek either to provide the results that researchers expect (compliance) or to make their actions appear socially desirable [35,36]. Considering the lower daily caloric intakes of both groups, calculated from self-reported dietary recalls, greater reductions in body mass should have been observed. Regardless, differences in compliance through WK4 between the groups is likely attributable to the daily ingestion of NOPE. However, the 50 mg per day lower dose used in this study compared to previous research [17], may have resulted in some degree of habituation following 4-weeks of supplementation, as previous investigations have noted dose-dependency [19,37].

It has been suggested that NOPE may curb appetite by slowing down gastric emptying [38] and intestinal motility [39]. While the present study did not measure gastric emptying and intestinal mobility directly, the lack of significant differences in caloric intake or feelings of satiety observed may reflect no differences in gastric emptying and intestinal mobility. Although both groups experienced reductions in caloric intake, possible differences may have been confounded by the participants’ physical activity habits. During lower intensity exercise (< 70% VO_2_max), caloric intake may increase due to stimulated gastric emptying [40,41], while more strenuous exercise may delay gastric emptying [41], thus decreasing appetite. Considering that exercise patterns were similar between the groups, it is possible that the potential influence of NOPE on appetite [38,39] likely enhanced dietary compliance through WK4.

The NOPE and EGCG combination has also been reported to reduce the severity of binge eating to a significantly greater degree than placebo [17]. While the present investigation noted decreases in BES scores for both groups, no significant differences were observed at any time point. However, baseline measurement scores suggest that neither group could be defined as binge eaters [34], which may imply that this proprietary supplement blend may be more effective in binge eaters. It is likely that reductions in BES scores in both groups may have also been the result of the combined diet and exercise program, as several studies have shown that a combination of diet and exercise may prevent the onset of binge eating in non-binge eaters [42,43].

The present investigation does support previous findings that the NOPE-EGCG blend can significantly enhance mood [17]. Oral supplementation of EGCG has been suggested to impact central nervous system function in the brain [44-46] by affecting γ-aminobutyric acid receptor binding sites (GABA_A_) [47]. Since γ-Aminobutyric acid is the principal inhibitory neurotransmitter receptor system in the brain that regulates anxiety, EGCG ingestion may reduce anxiety. EGCG was been shown to reduce anxiety in mice [48,49], while regular green tea consumption lowers post-stress cortisol levels and increases subjective relaxation in humans [50]. Our findings show that mood, feelings of depression and confusion are all improved after four weeks of supplementation, with improvements in feelings of tension thereafter.

## Conclusion

The results of this study indicate that a daily ingestion of a proprietary blend of 120 mg of NOPE and 105 mg of EGCG can enhance compliance to a low caloric diet, total mood score, feelings of fatigue, and confusion for 4-weeks, with improved feelings of tension thereafter. However, it does not have any significant effects on weight loss, changes in body composition, feelings of hunger, and binge eating in a group of healthy, overweight adults. The dose used in this study may have resulted in some degree of habituation following 4-weeks of supplementation.

## Abbreviations

BES: Gormally Binge Eating Scale; BMI: Body Mass Index; CB1: Cannabinoid type 1 receptors; EGCG: Epigallocatechin gallate; HSS: Hunger and Satiety Scale; KCAL: Kilocalories; NOE: N-oleyl-ethanolamide; NOPE: N-oleoyl-phophatidyl-ethanolamine; PLA: Placebo; POMS: Profile of Mood States; SUP: Supplement; TMS: Total Mood Score.

## Competing interests

The authors declare that they have no competing interests.

## Authors’ contributions

JRH was the primary investigator and responsible for study design. GTM was responsible for protocol supervision, patient recruitment, registration, data collection, data entry. JRH and JRS performed the statistical analysis, JRH, GTM, JRS, and MSF participated in manuscript preparation. AJW, AMG, NSE, WPM and TCS assisted with data collection and data analysis. All authors read and approved the final manuscript.
